# 46942 Risk factors, prevention, and screening practices for human papilloma virus associated cancers in Central-Eastern Puerto Rico

**DOI:** 10.1017/cts.2021.480

**Published:** 2021-03-30

**Authors:** Lilibeth Cruz-Martinez, Darleen Gonzalez-Galarza, Wilfredo E. De Jesus-Monge

**Affiliations:** Hospitales HIMA San Pablo

## Abstract

ABSTRACT IMPACT: The impact of this study is that the results may lead to the development of effective educational programs and a comprehensive cancer control program while verifying patients and medical care providers adherence and compliance with cancer clinical guidelines. OBJECTIVES/GOALS: The objective of this study is to assess risk factors, preventive measures, and screening practices for human papilloma virus (HPV) associated cancers in a sub-population in Central-Eastern Puerto Rico (PR). METHODS/STUDY POPULATION: This is a sub-analysis from an annual descriptive cross-sectional questionnaire of risk factors, preventive measures, and screening practices for cancer in PR administered at a private hospital campus using a convenience sample of healthy and non-healthy adults. RESULTS/ANTICIPATED RESULTS: Out of 345 enrolled subjects in 2019 for the questionnaire, 67 were enrolled by the first author, from which 66 (19%) subjects qualified for this sub-analysis for completing the study: 79% females. When analyzing HPV risk factors, 5% of the participants were smokers. Eleven percent of the subjects received the preventive HPV vaccine. Among those non-vaccinated and eligible for vaccination, 95% were willing to get it. Seventy one percent of females 21-29 years old and 97% of 30-65 years olds had age-appropriate cervical cancer screening. DISCUSSION/SIGNIFICANCE OF FINDINGS: Despite the low prevalence of HPV vaccination, almost all of the subjects within the age range for HPV vaccination were willing to get it. Also, there was a lower prevalence of cervical cancer screening in females 21-29 years old when compared with 30-45 years old. In conclusion, there is a need for more education about HPV associated cancers and vaccine.

